# Visceral Adiposity and Diet Quality Are Differentially Associated With Cognitive Abilities and Early Academic Skills Among Preschool-Age Children

**DOI:** 10.3389/fped.2019.00548

**Published:** 2020-01-14

**Authors:** Naiman A. Khan, Corinne Cannavale, Samantha Iwinski, Ruyu Liu, Gabriella M. McLoughlin, Linda G. Steinberg, Anne M. Walk

**Affiliations:** ^1^Department of Kinesiology and Community Health, University of Illinois, Champaign, IL, United States; ^2^Neuroscience Program, University of Illinois, Champaign, IL, United States; ^3^Division of Nutritional Sciences, University of Illinois, Champaign, IL, United States; ^4^Department of Human Development and Family Studies, University of Illinois, Champaign, IL, United States; ^5^Department of Kinesiology, Iowa State University, Ames, IA, United States; ^6^Department of Psychology, Eastern Illinois University, Charleston, IL, United States

**Keywords:** neuropsychology, nutrition, abdominal obesity, childhood, intelligence

## Abstract

**Background:** Visceral adipose tissue (VAT) and diet quality influence cognitive health in preadolescents; however, these relationships remain understudied among preschool-age children.

**Objectives:** Investigate the relationship between VAT, diet quality, academic skills, and cognitive abilities among preschool-age children.

**Methods:** Children between 4 and 5 years (*N* = 57) were enrolled in a cross-sectional study. Woodcock Johnson Early Cognitive and Academic Development Test (ECAD™) was utilized to assess General Intellectual Ability, Early Academic Skills, and Expressive Language. DXA was used to assess VAT. Diet quality was measured using the Healthy Eating Index-2015 (HEI-2015) based on 7-day food records.

**Results:** Greater VAT was associated with poorer Early Academic Skills (*r* = −0.28, *P* = 0.03) whereas a diet pattern that included Fatty Acids, Whole Grains, Saturated Fats, Seafood and Plant Proteins, Total Vegetables, and Dairy was positively associated with General Intellectual Ability (*r* = 0.26, *P* = 0.04).

**Conclusions:** Higher VAT is negatively related to Early Academic Skills whereas diet quality was positively and selectively related to intellectual abilities among preschool-age children. These findings indicate that the negative impact of abdominal adiposity on academic skills is evident as early as preschool-age while providing preliminary support for the potentially beneficial role of diet quality on cognitive abilities in early childhood.

## Introduction

Childhood is a period of dynamic brain growth and cognitive development that is partially shaped by a child's nutritional exposures (1). However, nutritional recommendations for children's brain health and cognitive function are absent from the U.S. Dietary Guidelines (2). This is concerning as the vast majority of children in the United States habitually fail to adhere to their recommended dietary guidelines i.e., exhibit poor diet quality (3). Currently, 1 in 3 children in the United States has overweight/obesity (≥ 85th BMI-for-age %ile) (4), which is concerning because excess fat mass or adiposity contributes to chronic disease as well poorer cognitive health (5, 6). Specifically, visceral adipose tissue (VAT), an adipose depot site well recognized for its detrimental metabolic implications, has been shown to predict poorer cognitive function in preadolescent children as well as adults (7–9). Therefore, there is an increasing need to understand the impact of diet and adiposity in predicting children's early academic skills and cognitive abilities.

Defining the influence of nutrients, foods, and diet patterns on cognitive abilities and early academic skills has become increasingly important based on an emerging body of literature demonstrating detrimental effects of nutrient deficiencies on both global and selective domains of cognition (1, 10). Executive function is a particular area of interest because it encompasses processes that underlie goal-directed behavior and are orchestrated by activity within the prefrontal cortex (11, 12). These core executive function processes (i.e., working memory, cognitive flexibility, and inhibitory control) build the foundation for reasoning, problem solving, and planning (13, 14). Executive function supports mental and physical health; success in school and in life; and cognitive, social, and psychological development (15). For example, in a large prospective study, Moffitt et al. (16) found that children who at ages 3–11 had better executive function were less likely to have overweight status, high blood pressure, or substance abuse problems as adults (16). Thus, given the long-term implications of executive function for scholastic success and well-being, it is important to determine how diet and adiposity are related to children's skills for these core cognitive control processes and early academic abilities.

Although executive function and academic skills develop gradually throughout childhood, there is a dearth in knowledge regarding the influence of habitual diet quality and adiposity on these abilities in early childhood. Virtually all the extant literature has focused on children of school age or children 6 years and older. For example, previous work by our laboratory has shown that preadolescent children with increased abdominal adiposity exhibit poorer performance on standardized academic tests and poorer ability for hippocampal-dependent relational memory (7, 8). Further, school-aged children (7–9-year-olds) with greater adherence to the recommended Dietary Guidelines of Americans, as assessed by the Health Eating Index-2005 (HEI-2005), exhibited greater cognitive control, even after adjusting for adiposity (17). In a larger study involving over 5,000 school-aged children, Florence et al. observed that children with greater diet quality, as assessed by the Diet Quality-International Index exhibited greater academic performance (18). Other studies have focused on populations with nutritional insufficiencies such as iron and vitamin deficiencies and their influence on academic achievement and/or cognitive performance. In a large review on the topic, Taras et al. (19) observed that food insufficiency is a serious problem affecting children's ability to learn, but its relevance to US populations needs to be better understood (19). Importantly, among the 25 studies, none were conducted among children younger than 5 years of age. This is concerning since the preschool age represents a key developmental stage where children begin to learn how to manage emotions, develop social skills, and self-regulation (20). Therefore, an important gap in the literature exists pertaining to the influence of dietary habits in early childhood and cognitive function and early academic achievement. Accordingly, the present work examined the cross-sectional relationships between adiposity, habitual diet quality, and early academic achievement and cognitive abilities using standard neuropsychological tests of early abilities for executive function and academic skills, among pre-school-aged children. We formed a directional hypothesis, informed by previous research in older children, that lower VAT and higher diet quality will be associated with greater performance on the standard neuropsychological tests among 4 and 5-year-olds.

## Methods

### Participants and Study Design

Males and females between 4 and 5 years (*N* = 57) were recruited from the Champaign-Urbana area. Children were screened prior to participation and were excluded based on several factors including presence of attentional and developmental disorders (Attention-Deficit/Hyperactivity Disorder, Autism Spectrum Disorder, Down's Syndrome), uncorrected vision, and hearing loss. All participants provided verbal and/or written assent and their guardians provided written consent prior to enrollment in the study. All study procedures were approved by the University Institutional Review Board and conformed to the guidelines of the Declaration of Helsinki. Study procedures took place over 2 laboratory visits. During visit 1, participants completed informed consent forms and underwent anthropometric assessments. Additionally, parents/guardians completed surveys to report the child's health history as well family and child demographic information (i.e., age, sex, household income). During visit 2, participants completed the pencil and paper-based neuropsychological battery [Woodcock Johnson Early Cognitive and Academic Development Test (ECAD™)] for assessment of general intellectual abilities and early academic achievement.

### Weight Status and Adiposity Assessment

Participants' height and weight were measured, without shoes, using a stadiometer (model 240; Seca, Hamburg, Germany) and a Tanita WB-300 Plus digital scale (Tanita, Tokyo, Japan), respectively. Each measurement was taken three times and the average was used for analyses. BMI-for-age-percentile cut-offs from the CDC were used to determine weight status (21). Adiposity was assessed by dual-energy X-ray absorptiometry (DXA) using a Hologic Horizon W bone densitometer (software version 13.4.2, Bedford, MA, USA). Percent whole-body fat mass (%Fat) and VAT were estimated using the standard software measure. This estimated VAT has been shown to correlate (*r* = 0.92; *P* < 0.01) with computed tomography (CT)-determined VAT values (22).

### Diet Quality Assessment

Children's habitual dietary intake was assessed by a 7-day food diary completed by the parent/guardian on behalf of the child. The intake information was analyzed using the Nutrition Data System for Research software (NDSR; Nutrition Coordinating Center, Minneapolis, MN, USA). The 7-day food record was provided to parents at the conclusion of their first laboratory visit. Parents were asked to return the food record to the research staff when they returned for their second laboratory visit. Healthy Eating Index (HEI)−2015 scores were derived to assess overall diet quality (Total HEI-2015).

Additionally, principal component analyses (PCA) were conducted to determine whether specific dietary patterns explained variance in cognitive outcomes. PCA creates these components by categorizing food groups together based on their inter-correlations and variance within the sample (23). Each component describes a different dietary pattern, and this pattern can be described by interpreting factor loadings, which are the correlations between components and input variables. Large positive or negative factor loadings indicate which foods are more important within that component. Components with factor loadings >0.4 are considered strong, and negative factor loadings indicate inverse correlations (23–25). We analyzed the 13 nutrient/food group components that constitute the Healthy Eating Index score (HEI-2015). We derived dietary patterns with a varimax rotation; therefore, the dietary factors were rotated by an orthogonal transformation. The number of factors to extract was determined by examining the total variance, diagram of eigenvalues (the Scree plot), and the interpretability of the factors. Each factor loading represents the degree and direction of the correlation between the HEI category and the component group (23–25).

### Cognitive Assessment

Children completed the Woodcock Johnson Test of Early Cognitive and Academic Development (ECAD™). The ECAD™ is comprised of 10 tests: seven measuring cognitive abilities and three measuring academic achievement. The tests in the ECAD™ combine to form three broad clusters with composite scores including General Intellectual Ability-Early Development, Early Academic Skills, and Expressive Language. The clusters comprised multiple tests that were multifaceted in that children were asked to respond to various visual and auditory presentations. The tests used to derive composite scores and their corresponding cognitive domains are outlined in Table 1. All testing and scoring material is provided by the publisher including flip books, audio recordings, scoring software, and examiner's manual. All standardization procedures were followed and the assessment was administered by a trained research assistant. This test has been normed and shown to be valid and consistent with other known standardized neuropsychological tests among children (26).

**Table 1:** Description of the scope of the tests of early cognitive and academic development (ECAD™), combination of tests, and domains assessed.

**Cluster/composite**	**Test**	**Domain/function assessed**
General Intellectual Ability-Early Development	Memory for names	Associative memory/long-term retrieval
	Sound blending	Phonetic coding/auditory processing
	Verbal analogies	Comprehension knowledge and fluid reasoning
	Visual closure	Closure ability/visual processing
	Rapid picture naming	Cognitive and linguistic fluency/processing speed
Early academic skills	Letter word recognition	Letter and word identification/reading-writing ability
	Number sense	Quantitative knowledge/mental math
	Writing	Reading-writing ability/spelling
Expressive language	^a^Picture vocabulary	Oral language development and word knowledge
	^a^Sentence repetition	Short-term working memory

a *Tests also included in the General Intellectual Ability-Early Development cluster*.

### Statistical Analyses

Initial Pearson's bivariate correlations were conducted to determine relationships between demographic factors, adiposity variables (BMI-for-age %ile, %Fat, and VAT), diet quality (Total HEI-2015 and Principal Components), and ECAD™ clusters (General Intellectual Ability, Early Academic Skills) and their constituent tests. Subsequently, partial correlations were conducted to determine whether the relationships diet and ECAD™ variables persisted following adjustment of any pertinent demographic or adiposity observed to be correlated with the ECAD™ outcomes in the bivariate correlations. Statistical significance was set at *p* = 0.05. Statistical analyses were conducted in SPSS version 24 (IBM, Chicago, IL). One-tailed analyses were conducted given the a priori directional hypotheses.

## Results

### Participant Characteristics

Participant sample characteristics are summarized in Table 2. The sample was comprised of 4 and 5-year-olds (*N* = 57) with 40% females and 60% males. The majority (70%) of the participants had healthy weight status and 23% had overweight or obese weight status based on BMI-for-age %tile. Woodcock Johnson ECAD™ tests were successfully completed by 54 participants whereas 53 successfully completed the DXA scan and 53 returned diet records. Forty-four participants provided complete data for diet, ECAD™, and VAT.

**Table 2:** Descriptive summary of participant characteristics, diet, and task performance.

	**Mean**	**SD**
Sex, females/males	23/34	
Maternal level of education *n* (%)		
High school graduate *n* (%)	1 (2)	
Some college *n* (%)	10 (20)	
Bachelor's degree *n* (%)	14 (28)	
Advanced degree *n* (%)	25 (50)	
Age, years	5.0	0.6
BMI-for-age %ile	51.8	33.7
Underweight, *n* (%)	4 (7)	
Normal or healthy, *n* (%)	40 (70)	
Overweight, *n* (%)	4 (7)	
Obese, *n* (%)	9 (16)	
Whole body fat, %	30.1	6.4
Visceral adipose tissue, *g*	111.9	67.7
Total Healthy Eating Index-2015	54.2	14.2
Total fruits	3.4	1.6
Whole fruits	4.0	1.5
Total Vegetables	2.0	1.2
Greens and beans	1.2	1.5
Whole grains	4.2	1.1
Dairy	2.8	1.9
Total protein Foods	4.9	3.2
Seafood and plant proteins	8.1	2.4
Fatty acids	3.5	2.8
Refined grains	4.6	3.4
Sodium	5.1	2.6
Added sugars	4.9	4.0
Saturated fats	5.5	2.6
General Intellectual Ability, standard score	109.1	14.2
Memory for names	108.3	10.1
Sound blending	100.2	14.2
Verbal analogies	106.4	17.8
Visual closure	101.7	11.1
Rapid picture naming	107.0	12.9
Early academic skills, standard score	101.4	14.3
Letter word recognition	99.8	15.5
Number sense	111.1	15.5
Writing	98.3	14.3
Expressive language, standard score	110.3	16.0
^a^Picture vocabulary	111.0	11.6
^a^Sentence repetition	108.2	16.4

a *Tests also included in the General Intellectual Ability-Early Development cluster*.

### Diet Quality and PCA

The principal components extracted, their constituent diet quality markers, coefficients, and variance explained are outlined in Table 3. Five components within our sample contained Eigenvalues >1 and collectively accounted for ~73% of variance. After further analysis, we decided to fix the number of extractions to three components and to suppress any values below 0.50. This assisted data interpretation. This collectively accounted for ~55.6% of variance. Component 1 (Fatty Acids, Whole Grains, Saturated Fats, Seafood and Plant Proteins, Total Vegetables, and Dairy) explained 21.3% variance, Component 2 (Total Fruit, Whole Fruit, and Sodium) explained 18% variance, and component three (Refined Grains, Green Beans, Total Protein, Added Sugars) explained 16.3% of variance.

**Table 3:** Principal components and coefficients as extracted from 13 constituents of the Healthy Eating Index-2015.

	**Food/nutrient score**	**Coefficients**	**Variance explained**
Principal component 1	Fatty acids	0.80	21.3
	Whole grains	0.65	
	Saturated fats	0.61	
	Seafood and plant proteins	−0.60	
	Total vegetables	0.54	
	Dairy	0.53	
Principal component 2	Added sugars	0.90	18.0
	Total fruit	0.84	
	Sodium	0.52	
Principal component 3	Refined grains	0.87	16.3
	Green beans	0.68	
	Total protein	0.53	

### Bivariate Correlations

**Figure 1:**
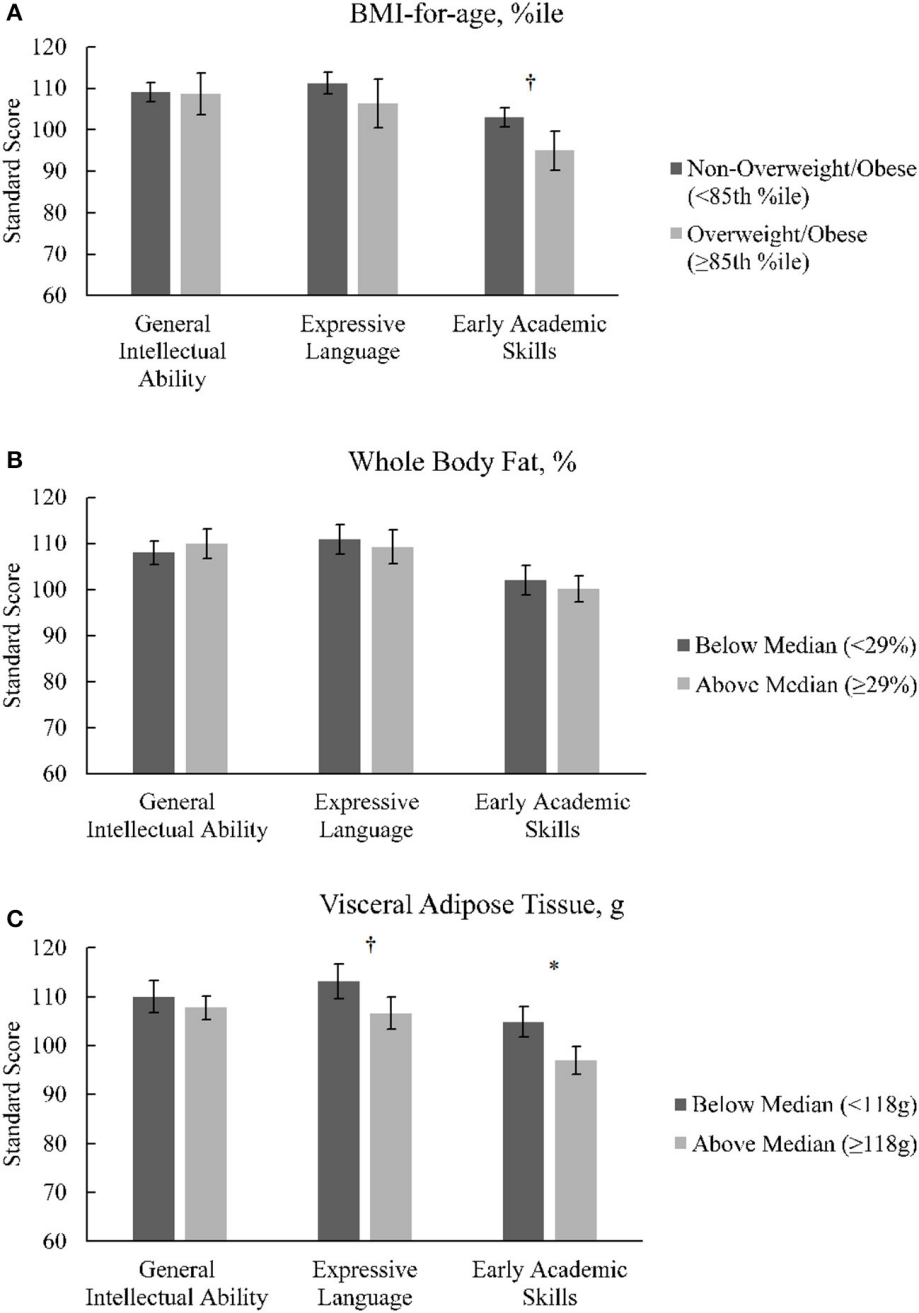
**(A–C)** Differences between bifurcated categories of BMI-or-age %ile, whole body adiposity (%Fat), and Visceral Adipose Tissue (VAT) across the main clusters of cognitive abilities and early academic skills. *p ≤ 0.05 (one-tailed) for independent *t*-test comparing categories for each cluster. ^†^*p* ≤ 0.10 (one-tailed) for independent *t*-test comparing categories for each cluster.

There were no significant correlations between demographic variables of age, sex, and household income and General Intellectual Ability. Similarly, there were no significant associations between Expressive Language and Early Academic Achievement and demographic variables (all *r*'s > 0.20, all *P*'s > 0.10). Correlations between adiposity variables and cognitive outcomes are described in Table 4. BMI-for-age %ile was inversely correlated with the Expressive Language cluster, possibly due to a marginally inverse influence on short-term working memory, as suggested by the trend-level relationship between BMI-for-age %ile and Sentence Repetition. Greater VAT was related to lower performance on the Early Academic Skills cluster, likely derived from poorer reading-writing ability among children with greater VAT, as indicated by the Letter Word Recognition and Writing tests. Additionally, VAT was inversely related to the Expressive Language cluster, primarily driven by the poorer oral language development among children with greater VAT, as assessed by the Picture Vocabulary test. On the other hand, there were no significant relationships observed for %Fat and the ECAD™ variables. For illustration purposes, the comparisons between bifurcated categories for the adiposity variables (i.e., BMI-for-age %ile, %Fat, and VAT) for the standard scores across clusters are presented in Figures 1A–C.

**Table 4:** Bivariate correlations between adiposity measures and neuropsychological outcomes.

		**BMI-for-age** **%ile**	**Whole body %Fat**	**Visceral adipose tissue**
General Intellectual Ability	*r*	−0.12	0.07	−0.11
	*P*	0.20	0.31	0.23
Memory for names	*r*	−0.01	0.01	0.10
	*P*	0.48	0.47	0.25
Sound blending	*r*	0.16	0.23	0.12
	*P*	0.13	0.06	0.21
Verbal analogies	*r*	−0.18	0.04	−0.19
	*P*	0.11	0.39	0.10
Visual closure	*r*	0.02	0.01	−0.08
	*P*	0.44	0.48	0.29
Rapid picture naming	*r*	−0.16	−0.19	−0.07
	*P*	0.12	0.10	0.32
Early academic skills	*r*	−0.14	−0.10	−0.28^*^
	*P*	0.17	0.24	0.03
Letter word recognition	*r*	−0.13	−0.08	−0.24^*^
	*P*	0.18	0.30	0.05
Number sense	*r*	−0.19	−0.11	−0.16
	*P*	0.09	0.23	0.14
Writing	*r*	−0.06	−0.08	−0.27^*^
	*P*	0.34	0.29	0.03
Expressive language	*r*	−0.27^*^	−0.16	−0.26^*^
	*P*	0.03	0.15	0.04
^a^Picture vocabulary	*r*	−0.08	0.11	−0.26^*^
	*P*	0.29	0.23	0.04
^a^Sentence repetition	*r*	−0.21	−0.05	−0.13
	*P*	0.07	0.36	0.20

* *Correlation is significant at the 0.05 level (1-tailed)*.

a *Tests also included in the General Intellectual Ability-Early Development cluster*.

Bivariate correlations between diet quality and cognitive function variables are described in Table 5. Diet Quality Principal Component 1 was positively associated with General Intellectual Ability and Expressive Language but not the Early Academic Skills cluster. The influence of Principal Component 1 on General Intellectual Ability was likely derived from the greater ability for auditory processing and fluid reasoning among children with higher intake of foods and nutrients included in Principal Component 1. Other tests found to be positively related to Principal Component 1 included sentence repetition, suggesting a beneficial role for short-term working memory. On the other hand, Component 2 was not associated with any of the three clusters. However, Principal Component 2 was associated with Picture Vocabulary test performance, indicating a relationship with oral language development. Principal Component 3 was related to Expressive Language cluster, possibly due to a trend-level benefit for short-term working memory, as indicated by the trend-level relationship with the Sentence Repetition test. Further, although Principal Component 3 was not significantly related to General Intellectual Ability, a positive relationship was observed for the Verbal Analogies test, suggesting a beneficial influence on fluid reasoning. Finally, Total HEI-2015 was positively related to the Expressive Language but not the Early Academic Skills and General Intellectual Ability cluster. Total HEI-2015 was positively associated with the Sentence Repetition test, suggesting a positive influence on short-term working memory. While the relationship between Total HEI-2015 and General Intellectual Ability was only evident at the trend-level, there was a positive association with performance on the Verbal Analogies test, indicating greater fluid reasoning among children with greater Total HEI-2015.

**Table 5:** Bivariate correlations between diet quality and neuropsychological performance.

		**Principal component 1**	**Principal component 2**	**Principal component 3**	**Total** **HEI-2015**
General Intellectual Ability	*r*	0.29^*^	0.21	0.14	0.22
	*P*	0.02	0.07	0.16	0.06
Memory for names	*r*	0.15	−0.04	−0.12	−0.03
	*P*	0.16	0.40	0.20	0.41
Sound blending	*r*	0.28^*^	0.19	0.11	0.23
	*P*	0.02	0.10	0.22	0.05
Verbal analogies	*r*	0.31^*^	0.12	0.28^*^	0.29^*^
	*P*	0.01	0.21	0.03	0.02
Visual closure	*r*	−0.12	<0.01	−0.17	−0.23
	*P*	0.21	0.49	0.12	0.06
Rapid picture naming	*r*	0.01	0.14	0.01	−0.01
	*P*	0.48	0.18	0.47	0.47
Early academic skills	*r*	0.10	−0.02	0.18	0.12
	*P*	0.26	0.45	0.11	0.21
Letter word recognition	*r*	0.17	−0.02	0.16	0.16
	*P*	0.11	0.46	0.13	0.14
Number sense	*r*	0.02	−0.10	0.11	−0.01
	*P*	0.46	0.25	0.23	0.48
Writing	*r*	−0.02	0.01	0.14	0.06
	*P*	0.45	0.49	0.16	0.34
Expressive language	*r*	0.26^*^	0.23	0.29^*^	0.29^*^
	*P*	0.04	0.06	0.02	0.02
^a^Picture vocabulary	*r*	0.16	0.27^*^	0.06	0.22
	*P*	0.13	0.03	0.35	0.06
^a^Sentence repetition	*r*	0.37^**^	0.18	0.21	0.307^*^
	*P*	<0.01	0.11	0.07	0.02

** *Correlation is significant at the 0.01 level (1-tailed)*.

* *Correlation is significant at the 0.05 level (1-tailed)*.

a *Tests also included in the General Intellectual Ability-Early Development cluster*.

*Component 1: Fatty Acids, Whole Grains, Saturated Fats, Seafood and Plant Proteins, Total Vegetables, and Dairy*.

*Component 2: Total Fruit, Whole Fruit, and Sodium*.

*Component 3: Refined Grains, Green Beans, Total Protein*.

### Partial Correlations

Given that VAT was related to a wider range of ECAD™ outcomes, we controlled for VAT in the partial correlations. Following adjustment of visceral adiposity, Component 1 was positively related to the General Intellectual Ability cluster (*r* = 0.26, *P* = 0.04). Specifically, greater score on Principal Component 1 was related to the Sound Blending test (*r* = 0.31, *P* = 0.02), demonstrating that the influence of Principal Component 1 on fluid reasoning was sustained, even after adjusting for VAT. The initially observed positive relationship between Principal Component 1 and the Expressive Language cluster was not sustained in the partial correlations; however, the relationship with short-term working memory, as indicated by greater performance on the Sentence Repetition test (*r* = 0.36, *P* < 0.01) persisted. Principal Component 2 was not associated with any cognitive variables. Principal Component 3 was positively related to the Verbal Analogies test (*r* = 0.25, *P* = 0.05). Finally, total HEI-2015 scores were inversely correlated with Visual Closure (*r* = −0.31, *P* = 0.02) and positively related to Sentence Repetition (*r* = 0.26, *P* = 0.05). However, the initially observed relationships between Total HEI-2015 and Expressive Language was not sustained following adjustment of VAT.

## Discussion

The influence of excess abdominal adiposity and diet quality on specific cognitive abilities and early academic skills remains understudied, particularly among pre-school aged children. The findings of the study revealed that specific dietary patterns, as identified by PCA, were selectively related to greater intellectual ability and early academic skills. This was particularly evident for the component that included consumption of Fatty Acids, Whole Grains, Saturated Fats, Seafood and Plant Proteins, Total Vegetables, and Dairy. Importantly, these relationships were sustained even after accounting for VAT. Specifically, the benefits of this pattern were selective for fluid reasoning and short-term working memory. Notably, the influence of diet quality was not evident for academic skills, suggesting that this relationship, previously observed in older children (17, 18), may emerge later in childhood. In addition to the relevance of diet quality for cognitive abilities and academic skills, the present study also extends the literature on accumulation of fat tissue in the visceral cavity and academic abilities by linking these factors among pre-school age children. Indeed, among adiposity variables examined, VAT was selectively and negatively associated with performance on multiple tests of reading and writing ability and oral language development. Given the previous work in older children and adults demonstrating links between abdominal adiposity and cognitive health, these findings provide support for the hypothesis that the detrimental influence of VAT on cognitive health emerge earlier in life. Collectively, the implications of the present study are that aspects of diet quality and VAT differentially influence cognitive abilities and academic skills in early childhood.

Considering the elevated prevalence of childhood obesity, an increasing body of work has demonstrated that excess fat mass negatively affects children's ability in several cognitive domains (27). Previous work by our team has demonstrated that weight status and the degree of adiposity is inversely related to children's performance on standardized academic achievement tests as well as tasks of memory, attention, and inhibitory control (7, 8, 28, 29). Specifically, VAT accumulation, in particular, appears to negatively impact children's cognitive function and academic achievement in both cross-sectional and prospective studies. For example, Scudder et al. (30) cross-sectionally linked greater waist circumference—a surrogate marker—of VAT to greater interference or poorer cognitive control among 2nd and 3rd grade children (30). Further, in a previous study by Raine et al. (31), 8–9-year-olds enrolled as waitlist control group participants in a 9-month daily physical activity intervention (FITKids) exhibited an increase VAT and the degree of increase in VAT was correlated with lower gains in cognitive control (31). The mechanistic links between VAT and cognitive health in children likely involve greater metabolic risk associated with adipose deposition in the viscera. VAT accumulation, surrounding internal organs such as the liver, is associated with type 2 diabetes (32–34), dyslipidemia (35), inflammation, increased risk of thrombosis (36, 37), and non-alcoholic fatty liver disease (38). Several factors differentiate VAT from other adiposity depots, including adipokine production, lipolytic capabilities, adipocyte size, and insulin sensitivity (39). However, virtually all the previous work linking fat distribution to cognitive function or academic achievement was conducted among older school-aged children. Therefore, the findings of the present work are novel because they link greater VAT to poorer Early Academic Skills. This revelation is concerning since it indicates that the link between VAT—a clinically pathogenic adipose storage site—and poorer cognitive health is evident earlier in childhood. Nevertheless, this work offers preliminary support for the beneficial influence of diet quality even after adjusting for VAT. Therefore, future intervention-based research is necessary to determine the impact of maintaining a high-quality diet on cognitive function in childhood.

Diet is thought to influence cognitive function by a variety of mechanisms that include, but are not limited to, providing essential nutrients for brain development (1), amelioration of neuroinflammation (40), and provision of energy (41). Given that the current literature on diet quality and cognitive function in early childhood is limited, the present work provides important evidence pointing to the potentially beneficial implications of healthy dietary patterns for cognitive abilities in early childhood. Previous work has shown that specific nutrients, such as iron, polyunsaturated fatty acids, and fiber, are related to greater academic achievement and cognitive function in children (42–45). However, dietary patterns analyses have greater translational potential since single nutrients do not typical exist in isolation and are likely to have synergistic or interactive effects for health (46). Limited research has previously examined the direct relationship between diet quality and academic achievement in children. For example, various diet quality indices including HEI and the Mediterranean-style diet assessed by KIDMED index were related to academic achievement in children and adolescents (17, 47). However, this work has focused on older children, resulting on little knowledge on the influence of diet on cognitive function in young children. To our knowledge, the present represents the first study to examine the influence of the degree of adherence to federally recommended Dietary Guidelines for Americans on cognitive abilities and academic skills among 4–5-year-olds. Additionally, we also employed a PCA approach that explored the potential influence of particular clusters or groups of food and nutrient components that may explain variability in cognitive abilities. These results indicated that, while total HEI-2015 scores were initially associated with cognitive abilities, this influence was not sustained for any major cluster following adjustment for VAT. On the other hand, a pattern that comprised of Fatty Acids, Whole Grains, Saturated Fats, Seafood and Plant Proteins, Total Vegetables, and Dairy was found to be robustly associated with General Intellectual Ability. Specifically, this pattern was beneficial for tests of auditory processing, fluid reasoning, and short-term working memory, even after adjusting for VAT. Thus, a select combination of foods and nutrients may have particular importance for cognitive abilities. Given that the focus of the current work was on diet quality, it is difficult to isolate the particular nutrients that may confer the cognitive benefits. However, several of the food groups and nutrients that would be more prevalent in component 1 have been previously shown to positively impact cognitive health including saturated fatty acids (48), dietary fiber (17) and carotenoids from vegetables and other plant foods (49–51), and proteins and vitamin D to name only a few (52). Thus, future experimental work is needed to confirm whether consuming a diet pattern rich in these diet quality components would be beneficial for children's cognitive function in a prospective manner.

Of note, there were no significant relationships observed between Early Academic Skills and total HEI-2015 or any of the Principal Components. This is surprising given previous work indicating the benefit of higher diet quality for academic achievement among older children (18). Thus, it is possible that this relationship emerges later in childhood. It could also be that early academic skills, such as early literacy in reading and math, is highly dependent on experience at this period in life, prior to formal schooling. If a child's parents haven't introduced the child to sight words, for example, they will not recognize sight words regardless of how intellectually gifted the child might be. Thus, academic skills at this age are largely a reflection of the child's intrinsic abilities, but their experience. As children get older, this issue is counteracted as they are all exposed to these concepts in school. Thus, later academic achievement may reflect other internal loci, like motivation, conscientiousness, etc. that drive achievement in school. Further, previous work in older children suggests that the beneficial relationship between diet quality and academic achievement is often observed among children with risk for nutritional inadequacies. Given that our study sample was predominantly comprised of highly educated families, it is possible that we were unable to detect this effect due to smaller effect sizes in this demographic. Interestingly, we observed that the only major predictor for Early Academic Skills was VAT. Therefore, it is likely that academic skills in early childhood are more susceptible to the detrimental influence of excess adiposity and less sensitive to dietary quality. The findings from this study provide novel evidence indicating that diet quality explains some of the variability in cognitive function and academic skills among preschool-aged children.

Although the present work provides potential insights into the diet quality implications for children's cognitive health, there are several limitations to consider. First, these results are based on cross-sectional analyses, precluding us from drawing causal inferences. In the current interpretation it is assumed that cognitive outcomes were driven by the dietary behaviors; however, the opposite may be true, such that higher cognitive abilities may contribute to better dietary choices. Future longitudinal and/or intervention studies examining the effects of diet quality on cognitive function could provide definitive support for the directionality of the associations observed in the current study. Finally, without biological markers of nutrient status and brain health, we can only speculate on the possible mechanisms that may underlie our observations. Nevertheless, this study aimed to test whether the positive relationships between VAT, diet quality, and cognitive health on older children are evident among 4 and 5-year-olds. The results provide the basis to conduct future rigorous trials to confirm the importance of diet quality for cognitive function in early childhood.

## Data Availability Statement

The datasets generated for this study are available on request to the corresponding author.

## Ethics Statement

The studies involving human participants were reviewed and approved by the University of Illinois Institutional Review Board. Written informed consent to participate in this study was provided by the participants' legal guardian/next of kin.

## Author Contributions

NK and AW conceived of the study including design. CC, SI, RL, GM, and LS were responsible for data collection and data interpretation. All authors were involved in writing the paper and had final approval of the submitted and published versions.

### Conflict of Interest

NK has previously received honoraria from the National Dairy Council. The remaining authors declare that the research was conducted in the absence of any commercial or financial relationships that could be construed as a potential conflict of interest.
